# When Muscles Matter in SORD Neuropathy

**DOI:** 10.1093/jnen/nlag057

**Published:** 2026-08-19

**Authors:** Heike Kölbel, Andreas Hentschel, Corinna Preuße, May Tiet, Maike Dohrn, David Muhmann, Jelle van den Ameele, Lei Chen, Ulrike Schara-Schmidt, Joachim Weis, Rita Horvath, Alexander Mensch, Andreas Roos

**Affiliations:** 1 Department of Pediatric Neurology, Centre for Neuromuscular Disorders, University Duisburg-Essen, 45147 Essen, Germany; 2 Leibniz-Institut für Analytische Wissenschaften-ISAS-e.V., 44227 Dortmund, Germany; 3 Charité-Universitätsmedizin Berlin, Corporate Member of Freie Universität Berlin, Humboldt-Universität zu Berlin, and Berlin Institute of Health (BIH), Department of Neuropathology, Berlin, Germany; 4 Department of Clinical Neurosciences, School of Clinical Medicine, John Van Geest Centre for Brain Repair, University of Cambridge, Cambridge, UK; 5 Department of Neurology, Medical Faculty, RWTH Aachen University, 52074 Aachen, Germany; 6 Department of Neuropathology, Medical Faculty, RWTH Aachen University, 52074 Aachen, Germany; 7 Department of Neurology, Martin-Luther-University of Halle-Wittenberg, Halle (Saale), Germany; 8 Department of Neurology, BG-University Hospital Bergmannsheil, Ruhr University Bochum, 44789 Bochum, Germany; Heimer Institute for Muscle Research, BG-University Hospital Bergmannsheil, 44789 Bochum, Germany; 9 Department of Neurology, Medical Faculty and University Hospital Düsseldorf, Heinrich Heine University, 40225 Düsseldorf, Germany; 10 Brain and Mind Research Institute, Children’s Hospital of Eastern Ontario Research Institute, Ottawa, ON K1H 8L1, Canada

**Keywords:** SORD neuropathy, Denervation-induced atrophy, Mitochondrial stress, Skeletal muscle metabolism, Polyol pathway

## Abstract

Biallelic pathogenic variants in *SORD*, encoding sorbitol dehydrogenase, have recently been identified as a common cause of autosomal recessive axonal Charcot-Marie-Tooth disease (CMT2). While the primary pathology is attributed to peripheral nerve degeneration, recent evidence suggests direct involvement of skeletal muscle. In this study, we investigated muscle biopsies from four genetically confirmed CMT-*SORD* patients (one presented with marked CK elevation of 13,000 after physical exercise) using an integrative approach combining histopathology, electron microscopy, proteomics, quantitative PCR, and biomarker analyses. Histological evaluation revealed features of chronic denervation with grouped fiber atrophy, fiber-type grouping and central nuclei, with central nuclei representing a non-specific finding in neurogenic muscle atrophy. Ultrastructural studies demonstrated mitochondrial abnormalities and expansion of the sarcoplasmic reticulum. Proteomic profiling identified 220 significantly dysregulated proteins in CMT-*SORD* muscle compared to controls, including alterations in mitochondrial complex I components, redox enzymes, and metabolic regulators distinct from changes observed in other rare recessive CMTs due to pathogenic *GAN*- and *GDAP1* variants. Quantitative PCR validated increased levels of *NNMT, POSTN, TACO1*, as well as complement (*C2, C5, C9*) and immunomodulatory (*CD4, CD8, CD68, CD169*) factors, suggesting mitochondrial stress, compensatory metabolic activation and tissue remodelling. Despite mitochondrial vulnerability, results from the serum studies indicate that GDF-15 and FGF-21 do not appear to be suitable biomarkers for CMT-*SORD*. These findings demonstrate that SORD deficiency induces molecular and structural changes in skeletal muscle that extend beyond denervation, implicating impaired sorbitol metabolism, oxidative stress, and mitochondrial dysfunction as contributors to disease pathology. The combined neurogenic and intrinsic myopathic features of *SORD*-related CMT2 underscore the need for therapeutic strategies targeting both neuronal and muscular compartments.

## Introduction

Charcot-Marie-Tooth disease type 2 (CMT2) is a hereditary axonal peripheral neuropathy that typically presents with distal motor and sensory impairment and shows marked genetic and clinical heterogeneity [1]. A substantial proportion of patients, up to 50% in some cohorts, remain without a molecular diagnosis despite extensive genetic testing, including gene panels and whole exome sequencing [1], [2]. The discovery of biallelic pathogenic variants in the *SORD* gene has recently explained a significant fraction of 7-9% of previously unsolved cases, establishing *SORD* neuropathy as one of the most common forms of autosomal recessive CMT2 [3]. As a point of reference, pathogenic variants in *MFN2* account for approximately 10-30% of cases of axonal neuropathies [4], [5], [6], [7], [8]. *SORD* encodes sorbitol dehydrogenase, a key enzyme in the polyol pathway responsible for converting sorbitol to fructose. Loss-of-function variants result in intracellular sorbitol accumulation, which induces oxidative stress, metabolic dysregulation, and progressive axonal degeneration, mechanisms previously implicated in diabetic neuropathy [9]. The most prevalent pathogenic allele is the frameshift variant c.757delG, with a carrier frequency of 0.00437 in gnomAD v3 [3]. Additional missense and splice-site variants have also been identified, some of which had initially been overlooked due to their population frequencies. Most patients are sporadic, but genetic testing of unaffected parents typically reveals monoallelic variants, confirming a recessive mode of inheritance (e.g. [10], [11], [12]).

Clinically, *SORD* neuropathy is characterized by progressive distal sensorimotor neuropathy with onset typically in the second decade of life. Serum sorbitol levels serve as a reliable biomarker for diagnosis and variant pathogenicity assessment. A multicenter phase 2/3 clinical trial is currently underway to assess the efficacy of govorestat, an aldose reductase inhibitor targeting the upstream enzyme in the polyol pathway (NCT05397665).

While muscle weakness in CMT is traditionally attributed to denervation, emerging evidence suggests that skeletal muscle may also be directly affected in *SORD* deficiency. A case report described a patient with biallelic *SORD* variants and no other myopathy-related variants who exhibited myopathological features, including fiber size variability, type 2 fiber atrophy, fibrosis, and desmin-positive structures [13]. Additional patients with *SORD* variants have shown elevated serum CK, myopathic findings on EMG, and muscle MRI abnormalities indicative of primary muscle involvement [14]. Indeed, a study on patients with *SORD*-related distal hereditary motor neuropathy reported by Li and colleagues revealed both neurogenic and subclinical myogenic features, including fiber atrophy, and minimal vacuolization. Ultrastructural analysis demonstrated mitochondrial abnormalities. Proteomic profiling identified a marked deficiency in mitochondrial complex I-associated proteins, with corroborative metabolic data indicating impaired NADH-dependent respiration, supporting mitochondrial dysfunction as a central pathogenic mechanism in addition to decline in glycogen metabolic processes and fructose 1,6-bisphosphate 1-phosphatase activity [15]. Recently, Dohrn and colleagues reported on neuropathological findings in two morphologically analyzed muscle biopsies revealing pronounced neurogenic atrophy, consistent with a predominantly motor neuropathy. In this context, they proposed that the observed myofibrillar alterations should be interpreted as neurogenic target changes rather than indicative of a primary myofibrillar or protein-accumulation myopathy [16]. Nevertheless, occasional abnormalities of the sarcoplasmic reticulum and T-tubule system, together with the presence of a necrotizing component for instance suggests that CMT-*SORD* may also involve a secondary myopathic process [16]. This could account for the elevated CK levels observed in some patients, although this has been descried as a rather unspecific effect observed also in other subtypes of CMT [17]. However, a reliable distinction between primary denervation-induced changes and concomitant myopathy would require examination of a larger cohort.

This study investigates muscle biopsies from four CMT-*SORD* patients (including two involved in the study of Dohrn and colleagues, [16] to better characterize skeletal muscle vulnerability beyond denervation, using an integrated approach of muscle MRI, histopathology, electron microscopy, molecular profiling, and quantitative PCR in addition to ELISA-based biomarker studies. Together with previous findings, our study expands the phenotypic spectrum of *SORD* neuropathy and raise the possibility that metabolic dysfunction within skeletal muscle may contribute independently to disease pathogenesis.

## Materials & Methods

Table 1 summarizes the muscle biopsies used for each analytical approach. Muscle biopsies derived from patients 2 & 3 were already been part of a previous study [16]. Muscle biopsies were collected for diagnostic purposes prior molecular genetic diagnosis was made in these four patients. The study was reviewed and approved by the Ethics Committee of University Medicine Essen (19-9011-BO).

### Microscopic studies on muscle biopsies

Histological investigations were performed according to standard protocols. Electron microscopic studies were conducted as described previously [18].

### Proteomic profiling on skeletal muscle biopsies

Mass spectrometry-based protein quantitation was carried out on whole protein extracts of gastrocnemius lateralis muscles as described before [19]. Doing so, we included a total of five CMT-muscles (*SORD* n=3 (patients 1-3), *GAN* n=1 & *GDAP1* n=1; see table 1) in addition to four healthy controls matching with *SORD* patients and four matching with *GAN* and *GDAP1* patients, respectively. Each sample was measured in technical triplicates. For the comparative analyses, we first identified the significantly upregulated and downregulated proteins in each dataset (“Denervation vs. Control” and “SORD vs. Control”). These regulated protein sets were used to construct Venn diagrams in order to determine the degree of overlap between both conditions. For the volcano plots, protein abundances from each comparison were averaged, and log_2_ fold changes as well as corresponding p-values were calculated to visualize differential regulation. For the Proteomap representation, all significantly regulated proteins from each comparison were mapped to depict the functional distribution of altered pathways.

### Quantitative PCR studies on whole RNA extracts of muscle biopsy specimens

Quantitative PCR (qPCR) analyses were performed as previously described [20]. All target transcripts were analyzed in technical triplicates, with a reference gene included in each run as an internal control for normalization of relative expression levels. The expression of *SORD, LIMCH1, TACO1, NNMT, POSTN, C2, C5, C9, CD4, CD8, CD68*, and *CD169* was assessed.

### ELISA-based quantification of GDF-15 and FGF-21

Serum level of GDF-15 and FGF-21 were studied in a cohort of 10 CMT-*SORD* patients including three of the four patients (2, 3 & 4) for which muscle biopsies were examined in this study. Additionally,10 control sera (derived from healthy age-matched donors) were included. Samples were analyzed using the ELLA microfluidic system (Bio-Techne, Minneapolis, USA), according to the manufacturer’s instructions.

## Results

### Demographic, clinical, and genetic data of CMT-*SORD* patients

Four individuals (two female, two male) from unrelated families were identified with SORD-associated Charcot-Marie-Tooth (CMT) neuropathy. Three patients were of Caucasian origin (non- consanguineous) and one of Arabic descent from a consanguineous family. Age at onset ranged from 12 to 23 years, with initial symptoms characterized by distal lower-limb weakness, foot drop, gait instability, and exercise intolerance. One patient exhibited sensory symptoms, including paraesthesia and burning pain, while myalgia and fatigue were common findings across the cohort.

Disease course was generally slowly progressive; however, two patients exhibited a biphasic or relapsing course that initially suggested immune-mediated neuropathy. Deep tendon reflexes were normal or brisk in the upper limbs but absent at the ankles. Serum creatine kinase (CK) levels were elevated in two patients (from 800 up to 5,000 U/L). After hiking in warm weather conditions and engaging in horseback riding, the patient - who previously had a CK level of 800 U/L (patient 2 in Table 2) - reported myalgia in the thighs and lower back and exhibited a CK elevation to 13,000 U/L. All clinical symptoms and CK levels returned to normal within one week. Cerebrospinal fluid analysis was normal in most cases, with only one showing mild protein elevation. Nerve conduction studies (NCS) demonstrated predominantly axonal sensorimotor neuropathy with reduced amplitudes and preserved conduction velocities, occasionally accompanied by mild demyelinating features. Electromyography (EMG) and muscle biopsies revealed consistent neurogenic patterns with fibre-type grouping indicating chronic denervation.

**Figure 1:**
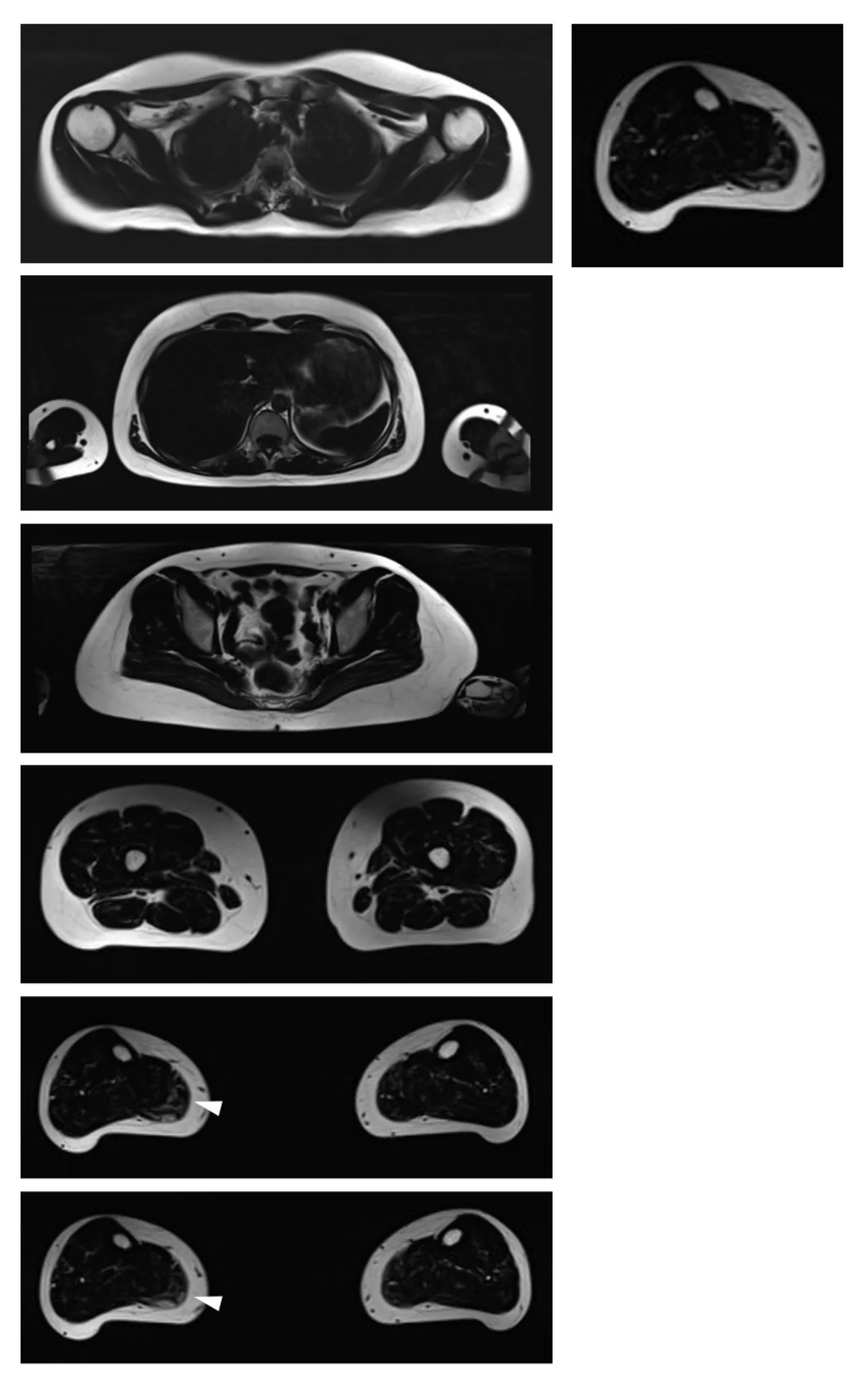
Muscle MRI findings in patient 4 Fat-weighted whole-body muscle MRI (Dixon imaging) of patient 4. Asymmetric fatty degeneration of the medial head of the gastrocnemius muscle is evident (white arrowhead; enlarged view in the right panel). The remaining musculature appears normal.

MRI of the lower limbs showed variable degrees of fatty replacement and mild oedema in calf muscles (Figure 1). Two patients received immunotherapies or pyridostigmine without clinical benefit. Our four cases were found to carry the most common pathogenic frameshift variant c.757delG (p.Ala253Glnfs*27) in exon seven of *SORD* (ENST00000267814.14) in a homozygous state. Collectively, these findings illustrated in tables 2 and 3, delineate a distinct phenotype of *SORD*-related axonal neuropathy, marked by adolescent onset, distal predominance, occasionally mildly to moderately elevated CK levels, and slowly progressive or fluctuating clinical course. A detailed clinical description for each of these four patients is presented in Supplementary Document 1.

### Histological findings

**Figure 2:**
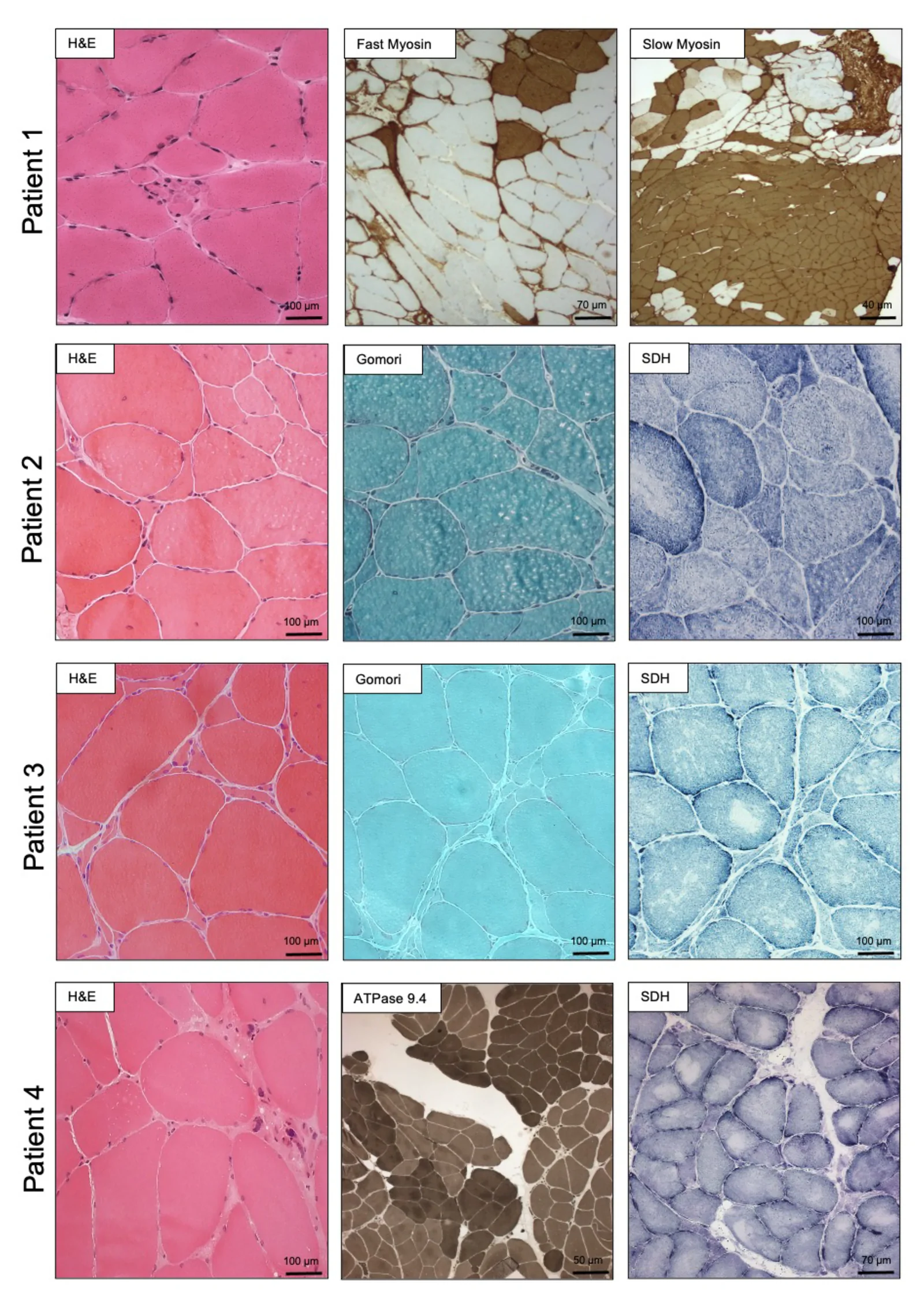
Histopathological features of skeletal muscle from CMT-*SORD* patients Hematoxylin and eosin (H&E) staining of muscle biopsies from four CMT-*SORD* patients reveals marked fiber size variation, group atrophy, angular fibers, and increased central nucleation. ATPase staining at pH 9.4, along with fast and slow myosin immunostaining, demonstrates fiber-type grouping, characterized by clusters of uniformly stained fibers, suggestive of collateral reinnervation. Succinate dehydrogenase (SDH) staining similarly shows fiber size variation and clustered fibers with irregular mitochondrial distribution and core-like lesions (targets). Representative images from each staining modality are shown.

In muscle biopsies from our CMT-*SORD* patients, H&E staining showed marked fiber size variation, groups of atrophic fibers, flattened and angular fibers and increased central myonuclei (within fibers of normal size or hypertrophic fibers). ATPase (pH 9.4) and fast as well as slow myosin stains revealed uniformly stained groups of fibers by means of fiber-type grouping, indicative of a reinnervation pattern. The SDH staining also showed marked fiber size variation with prominent presence of fibers with irregular mitochondrial distribution. These findings reflect chronic denervation and reinnervation, consistent with the motor predominant neuropathy. These findings are illustrated in figure 2. NADH stain supported the concept of irregular mitochondrial distribution but combined COX-SDH stain did not show any COX-negative fibers (Supplementary Figure 1A &B). There was indication of increased muscle fiber necrosis and myophagic activity in patient 2 who first presented with moderate CK elevation and significant CK increase (13,000 U/L) upon physical exercise. Notably, a muscle biopsy was obtained from this patient 13 years, prior for diagnostic purposes, before the significant CK elevation following physical exercise.

The myopathological findings of muscle biopsies from patients carrying genetically confirmed *GAN* and *GDAP1* variants have been described in previous studies [21], [22].

Immunohistochemical analyses were performed to further characterize the myopathology. These revealed a mild increase in immunoreactivity for C5b-9, CD45, and CD68 in the analyzed muscle biopsy of the pediatric patient 3 as well as in the adult patient 4 included in this study. In addition, a mild increase in CD3-positive cells was observed in the muscle tissue of the adult patient. It should be noted that tissue availability from the pediatric cohort was limited; consequently, only residual material from a single patient could be analyzed. Representative images of these immunohistochemical findings are shown in Supplementary Figures 1A and 1B. For comparison of immune cell activation, CD45, CD8, and CD68 stainings were also performed on skeletal muscle biopsies from juvenile ASyS patients, which demonstrated consistent immunoreactivity for these marker proteins. A representative case is presented in Supplementary Figure 1C.

Semithin sections of glutaraldehyde-fixed, resin-embedded muscle biopsy tissue stained with paraphenylenediamine revealed groups of partially atrophic, flattened or angular muscle fibers alongside hypertrophic fibers, as well as necrotic muscle fibers infiltrated by macrophages, consistent with the ultrastructural findings (Supplementary Figure 1D).

### Electron microscopic findings

**Figure 3:**
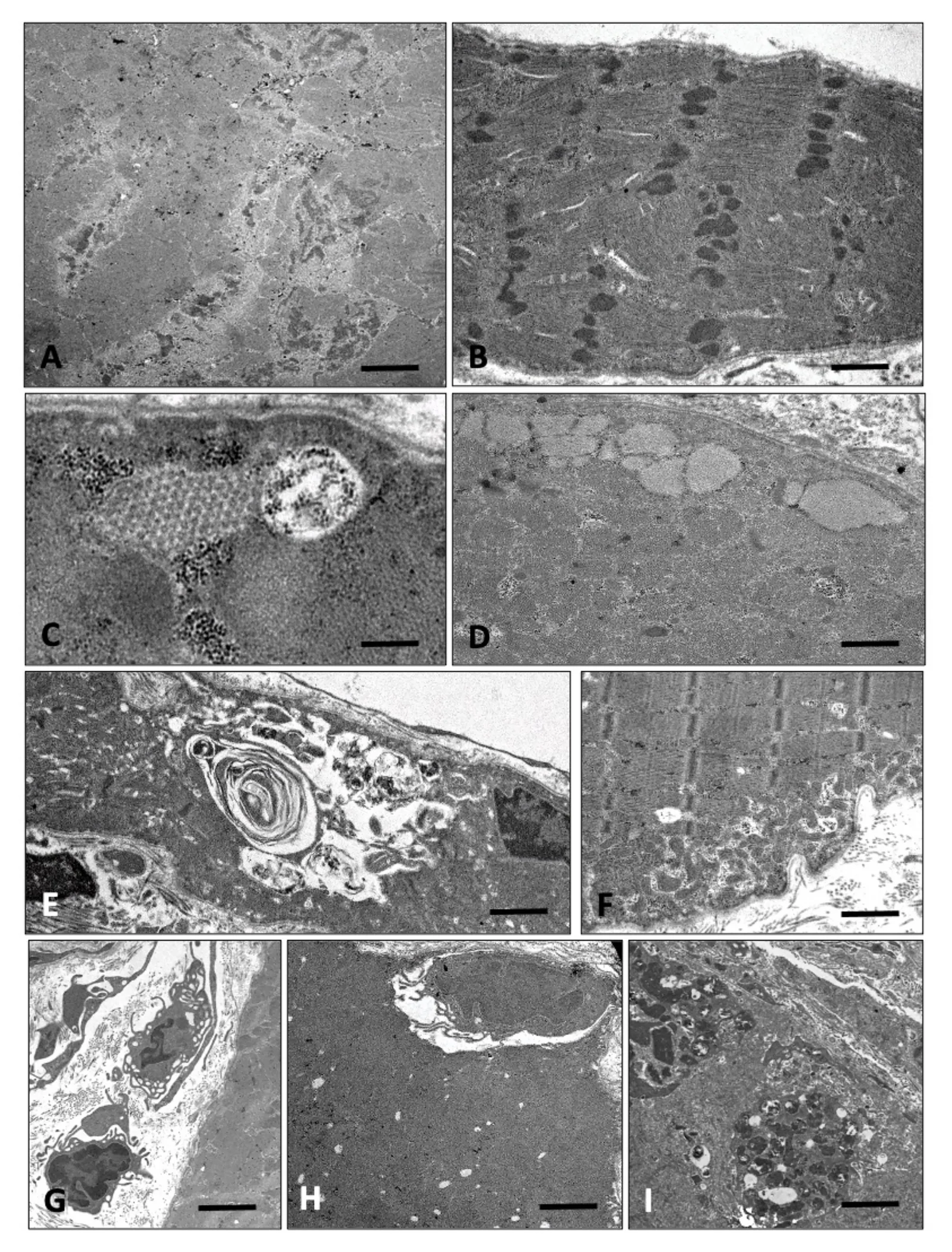
Electron microscopic findings in patients 2 & 3 (**A**) Z-band disintegration, cross section. Scale bar = 2.5 μm. (**B**) Nemalin body-like Z-band thickening. Scale bar = 1.0 μm. (**C**) Minor focal honey comb-type proliferation of T-tubules. Scale bar = 0.25 μm. (**D**) Moderate focal widening of the sarcoplasmic reticulum. Scale bar = 0.8 μm. (**E**) Large autophagic vacuole in an atrophic muscle fiber. Scale bar = 2.5 μm. (**F**) A slight accumulation of normal mitochondria beneath the sarcolemma. Insert: Enlarged view of the mitochondria shown below to illustrate the normal morphology of the cristae. Scale bar = 1.5 μm. (**G**) Activated monocytes travelling the endomysial space. Scale bar = 12 μm. (**H**) Macrophage invading a muscle fiber. Scale bar = 6 μm. (**I**) Debris-filled macrophages within a necrotic muscle fiber. Scale bar = 7.5 μm.

In atrophic fibers, there were myofibrillar alterations, including Z-band disintegration and occasional nemaline bodies (Figure 3A & B), most likely secondary to denervation. We observed few honeycomb-like structures, within normal limits (Figure 3C). Also, moderate dilatation of the sarcoplasmic reticulum (Figure 3D) as well as the scattered autophagic vacuoles (Figure 3E) were rarely seen, most likely ultra-structural consequences of muscle cell denervation. Mitochondrial number and morphology were unremarkable (Figure 3F). The most prominent finding was a pronounced monocyte/macrophage activation in both biopsy specimens; in patient 2, this was further accompanied by muscle fiber necrosis and myophagic reactions (Figures 3G-I).

### Proteomic profiling unravels protein changes characteristic in SORD-deficient muscle

**Figure 4:**
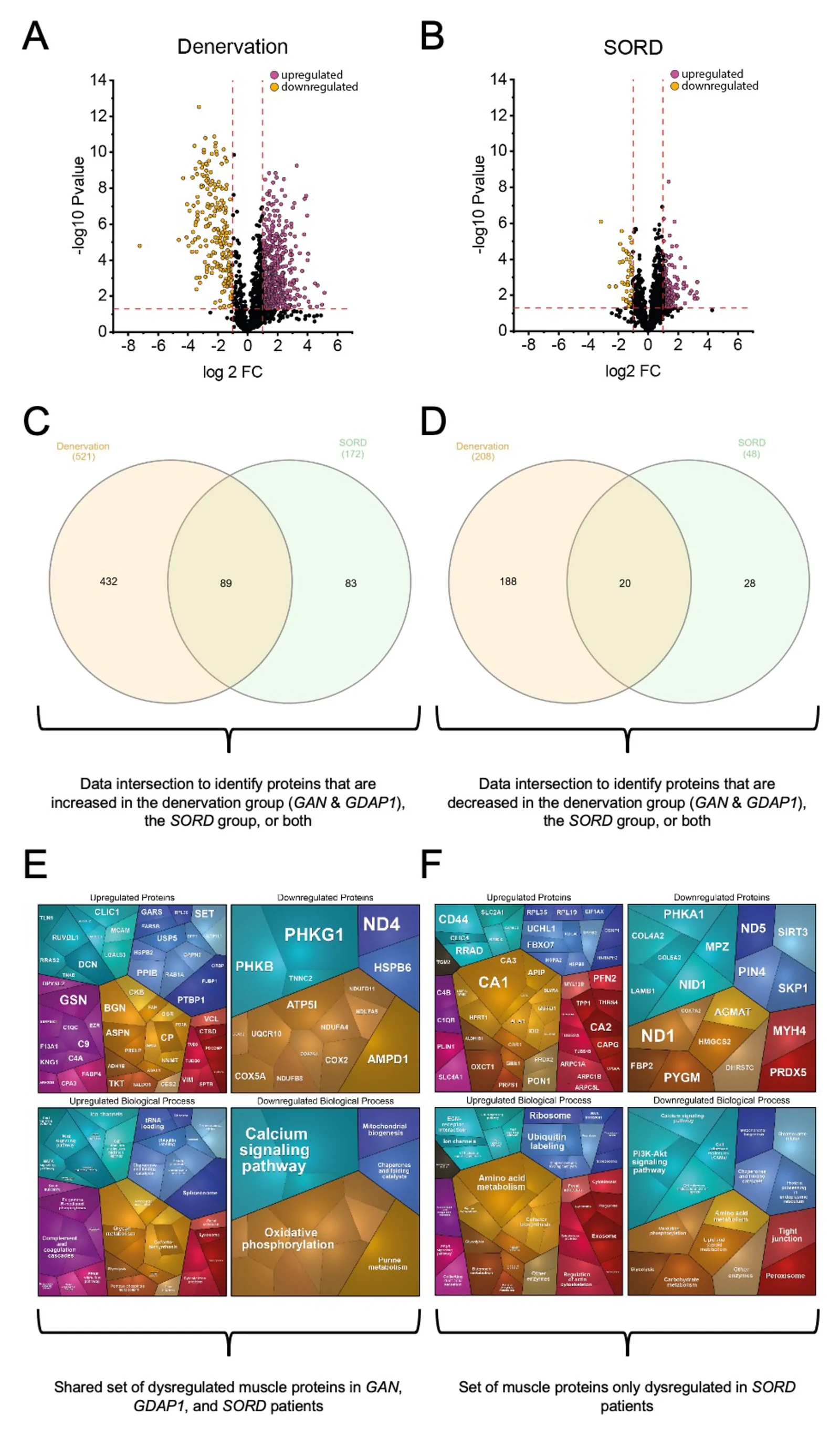
Comparative proteomic profiling reveals CMT-SORD–specific molecular alterations in skeletal muscle (**A**) Quantitative proteomic analysis of skeletal muscle from *GAN* and *GDAP1* CMT patients (denervation reference group) versus age-matched controls identified 729 significantly dysregulated proteins (adjusted *p* < 0.05), including 521 upregulated and 208 downregulated proteins. (**B**) Proteomic profiling of skeletal muscle from CMT-*SORD* patients revealed 220 significantly altered proteins compared to controls, with 172 upregulated and 48 downregulated. (**C**) Comparative analysis of CMT-*SORD* and disease control proteomes showed 432 proteins uniquely increased in the denervation group, 83 proteins exclusively increased in CMT-*SORD* muscle, and 89 commonly upregulated proteins. Among downregulated proteins, 188 were unique to the denervation group, 28 were specific to CMT-*SORD*, and 20 were shared. (**D**) Proteomaps-based pathway analysis of proteins commonly increased across all groups revealed enrichment in signalling cascades (e.g., WNT, MAPK), complement activation, mRNA splicing, cytoskeletal organization, and various metabolic pathways including glycolysis, amino acid, glycan, and pentose phosphate metabolism. Shared downregulated proteins affected calcium signalling, chaperone function, mitochondrial biogenesis, oxidative phosphorylation, and purine metabolism. (**E**) Proteins exclusively upregulated in CMT-*SORD* muscle were associated with PPAR, HIF-1, Ras, and ErbB signalling pathways, ECM-receptor interactions, complement activation, exosome function, cofactor biosynthesis, and multiple metabolic pathways (e.g., glycolysis, purine, lipid, and amino acid metabolism). (**F**) CMT-*SORD*–specific downregulated proteins impacted calcium and PI3K-AKT signalling, cell adhesion, chaperone function, protein synthesis, peroxisomal and mitochondrial function, and metabolic pathways related to carbohydrates, amino acids, lipids, and steroids. Together, these findings distinguish *SORD*-associated proteomic alterations from those driven solely by denervation, highlighting disease-specific molecular signatures in CMT-*SORD* skeletal muscle. See Supplementary Table 1 for complete protein lists.

To distinguish biochemical alterations due to denervation from those directly attributable to the loss of functional SORD in skeletal muscle, we first determined the proteomic signatures of muscles from *GAN* and *GDAP1* CMT patients (clinical reference group) by comparing their protein abundances to those in age-matched control muscles. We identified the significant dysregulation of 729 proteins (adjusted p < 0.05), including 521 increased and 208 decreased proteins quantified based on a minimum of two unique tryptic peptides (Figure 4A). The same approach was applied to the CMT-*SORD* patient group. Proteomic profiling of skeletal muscle tissue from CMT-*SORD* patients revealed distinct changes compared to controls, with a total of 220 proteins significantly dysregulated (adjusted p < 0.05), including 172 increased and 48 decreased proteins (Figure 4B). Subsequently, we intersected the proteomic data from the disease control group with that of the CMT-*SORD* group. This analysis identified 432 proteins uniquely increased in the disease controls, whereas 83 proteins were exclusively upregulated in the CMT-*SORD* group; 89 proteins were increased in both groups. In terms of downregulated proteins, 188 were decreased in the denervation group and 28 were uniquely decreased in the CMT-*SORD* group, with 20 proteins commonly decreased in both groups (Figure 4C and Supplementary Table 1). These findings suggest that, in addition to neurogenic muscle atrophy, *SORD* neuropathy is associated with characteristic molecular alterations in skeletal muscle. To focus on the different biological processes affected by respective protein dysregulations, a proteomaps-based in silico analysis [23] of proteomic data was conducted: The shared set of increased muscle proteins in *GAN, GDAP1* and *SORD* patients impact among others on different signaling cascades (including WNT & MAPK mediated cascades), activation of the complement system, splicing along with protein production and -turnover, cytoskeleton as well as amino acid, glycan and pentose phosphate metabolism in addition to glycolysis. The shared set of decreased proteins affect Ca^2+^ signaling, the chaperone system, mitochondrial biogenesis, purine metabolism and oxidative phosphorylation (Figure 4D). However, biological processes affected by proteins increased only in skeletal muscle of CMT-*SORD* patients include different signaling cascades (such as PPAR, HIF-1, Ras and ErbB cascades), extracellular matrix and receptor interaction, activation of the complement system, cytoskeleton and exosome function, protein synthesis and turnover, co-factor biosynthesis, glycolysis as well as purine, lipid and amino acid metabolism (Figure 4E). Focusing on the set of muscle proteins decreased only in CMT-*SORD* patients, data analysis revealed an impact also on signaling cascades (Ca^2+^ and PI3K-AKT cascades), cell adhesion and tight junctions, chaperone function along with protein synthesis and -turnover, peroxisomal function and glycolysis, carbohydrate and amino acid as well as lipid and steroid metabolism in addition to oxidative phosphorylation and mitochondrial biogenesis (Figure 4F).

### Quantitative PCR studies show decreased *SORD* transcripts and confirm molecular changes unveiled by proteomics

Quantitative PCR (qPCR) analyses were performed on whole RNA extracts from muscle biopsies under several premises. First, we investigated whether the c.757delG (p.(Ala253GlnfsTer27)) variant present in all patients exerts a comparable effect on the abundance of the corresponding transcript. Surprisingly, qPCR analyses revealed inconsistent results: while patients 3 and 4 each showed an 81% reduction in transcript levels, the reduction in *SORD* expression in patient 2 was only 46%. Expression of *LIMCH1*, which encodes a marker protein associated with target fiber formation and reinnervation in peripheral neuropathies [24], was increased by 135% and 136% in patients 2 and 4, respectively, and by 32% in patient 3. In addition, a selection of transcripts was analyzed to validate the proteomic data. *TACO1* expression was increased by 145% in patient 2, 8% in patient 3, and 125% in patient 4. A marked upregulation of *NNMT* was observed, with a 5275% increase in patient 2, compared to 219% in patient 3 and 389% in patient 4. *POSTN*, encoding a protein involved in fibrotic remodeling of skeletal muscle [25], showed increases of 1990% in patient 2, 3260% in patient 3, and 840% in patient 4. Analysis of complement factors to further corroborate the proteomic findings revealed increased expression of *C2* (1126%, 947%, and 340%), *C5* (340%, 84%, and 269%), and *C9* (454%, 302%, and 754%) in patients 2, 3, and 4, respectively. To further validate the proteomic data, immunological markers were also assessed at the transcript level. This demonstrated increased expression of *CD169* (3230%, 644%, and 414%), *CD4* (3201%, 722%, and 264%), *CD68* (2802%, 551%, and 280%), and *CD8* (3465%, 695%, and 150%) in patients 2, 3, and 4, respectively. These transcriptomic data are summarized in Figure 5. Overall, patient 2 - who exhibited the least reduction in *SORD* expression - consistently showed the strongest upregulation of transcripts associated with myopathological processes, suggesting the most pronounced molecular pathology. This is particularly evident in comparison to patient 3, another pediatric case, who displayed the highest *POSTN* levels, indicative of pronounced fibrotic remodeling. patient 4, although not included in the proteomic analyses, also showed consistent upregulation of transcripts used for proteomic validation. This supports the robustness of the mass spectrometry data and suggests a characteristic protein signature of myopathology in CMT-*SORD*. These qPCR findings alre illustrated in Figure 5.

### ELISA-based protein quantitation studies of FGF-21 and GDF-15 as serum biomarkers for CMT-*SORD*

**Figure 6:**
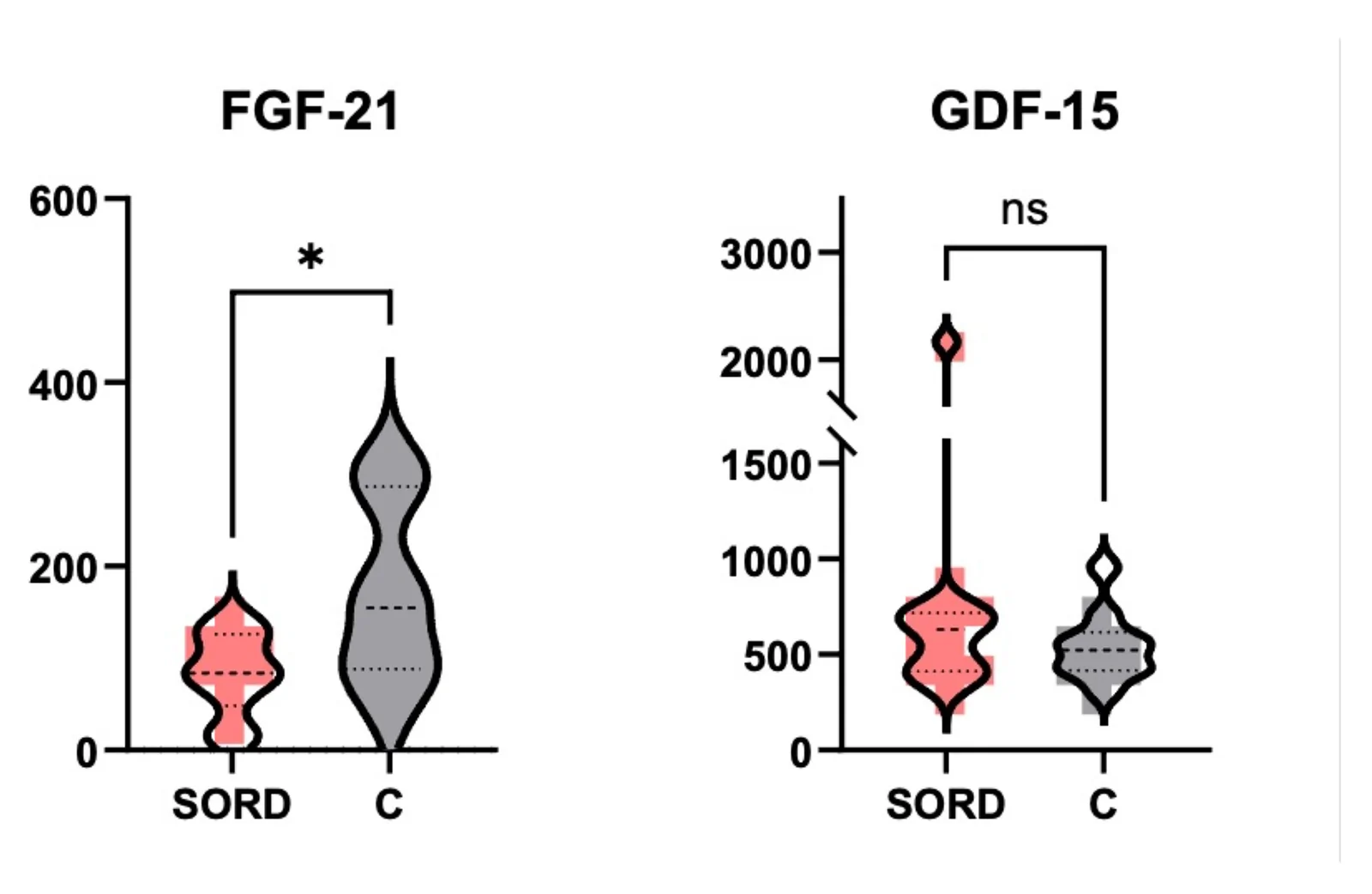
Circulating levels of FGF-21 and GDF-15 in SORD patients and controls Serum concentrations of FGF-21 (left) and GDF-15 (right) were measured by Enzyme-Linked Immunosorbent Assay (ELISA) in individuals with CMT-*SORD* and healthy controls (C). Each point represents an individual participant, and horizontal lines indicate mean ± SEM. FGF-21 levels were significantly lower in the CMT-*SORD* group compared with controls (p < 0.05). No significant difference (n.s.) was observed between groups for GDF-15.

ELISA-based quantification of GDF-15 did not show significant changes in comparison to the healthy control group (Figure 6). In contrast, FGF-21 levels were significantly lower in the CMT-SORD group compared with controls (p < 0.05) (Figure 6).

## Discussion

In 2020, Cortese and colleagues identified bi-allelic variants in *SORD* as the genetic cause of autosomal recessive distal hereditary motor neuronopathy type 8 (HMNR8; OMIM 618912), also referred to as *SORD* deficiency with peripheral neuropathy (SORDD) [3]. Since then, multiple studies have corroborated the association between pathogenic *SORD* variants and peripheral neuropathy (e.g. [10], [11], [12], [26], and early therapeutic strategies have been initiated [27].

More recently, three independent reports have explored the impact of SORD deficiency on skeletal muscle. A key challenge in interpreting these findings lies in disentangling morphological and biochemical changes resulting from denervation from those directly attributable to the loss of functional SORD within muscle cells. This distinction is particularly complex, because there is evidence that functional SORD may be required during denervation, potentially leading to secondary pathophysiological effects in skeletal muscle under such conditions. Under normal physiological circumstances, skeletal muscle primarily utilizes glucose and fatty acids for energy, and sorbitol is not a major metabolite. While small amounts of sorbitol can be produced via the polyol pathway - through aldose reductase-mediated conversion of glucose to sorbitol, followed by conversion to fructose via sorbitol dehydrogenase - this pathway is typically less relevant under normoglycemic conditions. However, in certain cell types, including skeletal muscle, this pathway can become upregulated under stress, such as hyperglycaemia or altered muscle activity. Denervation and chronic low-frequency stimulation are known to induce structural, physiological, and biochemical remodelling. For example, studies in rabbit models have shown that denervation leads to a significant increase in fructose 2,6-bisphosphate concentrations within 48 hours [28], highlighting a potential metabolic shift. These findings raise the possibility that SORD deficiency may interfere with such adaptive metabolic responses during denervation, thereby contributing to muscle pathology. However, it is noteworthy that in amyotrophic lateral sclerosis, a motor neuron disorder, muscle pathology arises not solely from denervation but also from mutant protein expression and subsequent intrinsic pathophysiological cascades within muscle cells, which contribute to progressive weakness and disrupt the integrity of neuromuscular junctions [29], [30]).

In this study, we combined microscopic and proteomic analyses to investigate the morphological and biochemical consequences of functional SORD loss in skeletal muscle. Building on the recent study by Li et al., which included a proteomic analysis of skeletal muscle from CMT-*SORD* patients (n = 5) [15], we expanded the investigation by incorporating additional biopsies from patients with other neuropathy-associated genotypes. To delineate protein alterations characteristically attributable to SORD deficiency from those resulting from denervation itself, we compared muscle proteomic profiles of three genetically confirmed CMT-*SORD* patients with denervated muscle biopsies of patients carrying *GAN* and *GDAP1* mutations, which served as clinical references for denervation-associated changes. This comparative approach identified 89 proteins consistently upregulated and 20 consistently downregulated across all three genotypes, representing a shared molecular signature of denervation. Importantly, our analysis also revealed a distinct set of 83 proteins upregulated and 28 proteins downregulated exclusively in the CMT-*SORD* cohort, suggesting characteristic molecular consequences of SORD deficiency that extend beyond general denervation-related changes. Quantitative PCR validation studies, including an additional muscle biopsy from a CMT-*SORD* patient (patient 4), confirmed the proteomic findings - including increase of factors of the complement cascade and immunoregulatory proteins - showing robustness of our biochemical findings. These targeted qPCR analyses further demonstrated increased expression of *LIMCH1*, which encodes a marker of target fiber formation in muscle from patients with peripheral neuropathy [24], thereby providing a molecular correlate to the irregular mitochondrial distribution observed histologically, most consistent with the presence of target fibers.

Our integrative analysis reveals that SORD deficiency profoundly affects skeletal muscle homeostasis, with dysregulation of mitochondrial proteins emerging as a central pathomechanism. Proteomic data showed a marked decrease in mitochondrial proteins, a pattern that was also identified as a common consequence of muscle denervation through our intersection with proteomic data from other denervation-related muscle diseases. This finding aligns well with previous literature describing mitochondrial protein loss as a hallmark of denervated muscle [31]. Based on proteomic profiling, we identified a decrease in cytochrome c oxidase and other cytochrome-c-modulating proteins which may reflect mitochondrial stress, impaired electron transport, or early stages of apoptosis, where cytochrome c itself is increased or released into the cytosol [32]. However, electron microscopy demonstrated normal mitochondrial morphology in *SORD*-mutant muscle of patients 2 & 3. This ultra-structural finding is in contrast with the morphological observation of perturbed mitochondrial architecture as recently reported by Li and colleagues [15]. Although ultra-structural analysis revealed normal mitochondrial morphology in our study, the identified proteomic alterations likely reflect functional or regulatory disturbances - potentially early, compensatory, or denervation-associated - that do not necessarily translate into ultrastructural abnormalities detectable by electron microscopy. This interpretation is further supported by one of our previous studies [33], which demonstrated proteomic dysregulation indicative of metabolic reprogramming and alterations in lipid profiles, in the absence of detectable changes in mitochondrial morphology, in skeletal muscle from a *FAR1* patient presenting with generalized muscle weakness accompanied by atrophic and angular fibers.

Consistent with the preserved mitochondrial structure and the notion of biochemical compensation, circulating GDF-15 levels, a biomarker of mitochondrial stress, remained unchanged in CMT-*SORD* patient serum. In contrast, FGF-21 levels were reduced, which is in contrast to increased FGF-21 in mitochondrial myopathies due to impaired mitochondrial translation [34]. However, closer inspection of the absolute values and the corresponding plot indicates that, although statistically significant, the difference is unlikely to be biologically or clinically relevant. These observations therefore suggest that FGF-21 levels are not substantially altered in individuals with SORD deficiency. In silico studies of our proteomic data unveiled a substantial overlap in the biological processes affected by proteins that are commonly dysregulated in both groups and those altered exclusively in the CMT-*SORD* group. This may indicate that while the same core biological processes are involved based on denervation, their more pronounced downregulation in the CMT-*SORD* group could have a greater impact on the functional vulnerability of these pathways. This hypothesis is supported by the observation that several metabolic cascades (such as carbohydrate metabolism, lipid metabolism, and peroxisomal function) appear to be more prominently affected in the CMT-*SORD* group compared to the shared group. While SORD is directly involved in carbohydrate metabolism via the polyol pathway, the observed alterations in lipid metabolism and peroxisomal dysfunction are more likely secondary effects. In SORD deficient states, the accumulation of reactive oxygen species (ROS) and disruption of the NAD^+^/NADH balance may impact membrane lipid composition or promote lipid peroxidation, thereby contributing to these secondary metabolic disturbances. Along this line, disrupted NAD^+^/NADH balance leads to cellular oxidative stress burden, which in turn drives mitochondrial dysfunction and triggers compensatory responses. This pathophysiological interplay is reflected in the dysregulation of cytochrome proteins and upregulation of ALAD, an enzyme in tetrapyrrole (heme) biosynthesis. Numerous antioxidant and redox defence proteins were increased in patient muscle, including AFM, ALDH1A1, BLVRA, CA1 & 3, CBR1, CLIC4, GSTO1, GSTZ1, PON1, PRDX2, TGM3, and TXNDC17, indicating activation of protective pathways against oxidative damage and by the same token highlighting oxidative stress burden as a major pathophysiological driver in myopathology underlying SORD deficiency. Beyond redox regulation, alterations in nucleotide metabolism were evident, as shown by increased abundance of HDDC2 and NME2, suggesting adaptive responses to NAD^+^ depletion and increased nucleotide turnover. An adaptive response to NAD^+^ depletion is moreover supported by increase of NNMT acting as a key metabolic-epigenetic regulator by catalyzing the methylation of nicotinamide, thereby linking one-carbon metabolism to NAD^+^ levels. Notably, NNMT inhibition has been shown to enhance exercise-induced improvements in muscle function [35], suggesting that its upregulation in CMT-denervated muscle as shown in our study may contribute adversely to the decline in muscle performance. IDI2, a muscle-enriched enzyme in the mevalonate pathway, was also upregulated, likely reflecting metabolic shifts in isoprenoid and lipid biosynthesis, consistent with the observed increase in PLIN1 and 3. Importantly, oxidative stress and mitochondrial dysfunction may also contribute to immune activation and inflammation, known drivers of muscle atrophy in denervation and metabolic disease. Muscle biopsies showed macrophage infiltration ([16] and CD68 stain of this study), and proteomics identified upregulation of Macrophage-capping protein and multiple immune-related proteins including immunoglobulins, complement factors, and ITIH4. ITIH4 emerged as a biologically plausible regulator of muscle inflammation due to its established roles in ECM stabilization, protease inhibition, and macrophage modulation [36], [37]. HPRT1, increased in muscles of our CMT-*SORD* patients (Supplementary Table 2), was recently described as massively increased in myositis after anti-CD30 therapy [38]. These findings were corroborated by immunostaining, which confirmed B-cell and complement activation and are in line with our electron microscopic observations. This inflammatory activity may also contribute to the proteomic changes involving peroxisome-associated pathways.

A metabolic shift in SORD deficient muscle, potentially driven by the increased demand for fructose synthesis during denervation, is supported by the recent finding of decline of FBP1 activity in CMT-*SORD* patient muscles [15] and moreover reflected in our proteomic signature by the downregulation of several key proteins. Notably, DHRS7C, an NADH-dependent oxidoreductase that catalyzes the oxidation of all-trans-retinol to all-trans-retinal, is decreased. Overexpression of DHRS7C in skeletal muscle has been shown to activate mTORC2, enhance glucose metabolism, and improve muscle performance [39], suggesting that its reduction may contribute to altered glucose metabolism and impaired muscle function in SORD deficiency. Further evidence of disrupted glucose-to-fructose conversion is indicated by decreased levels of FBP2. Impaired glycogen metabolism is supported by the reduced abundance of PYGM and the PHKA1. Additionally, several proteins uniquely dysregulated in CMT-*SORD* patient muscles may have direct pathophysiological relevance, including increase of CD44, a cell surface glycoprotein that binds sorbitol-modified hyaluronic acid (HA/sorbitol) and mediates various cell survival, adhesion, migration, and inflammatory responses [40]. Recent evidence suggests a role of CD44 in metabolism regulating diet-induced insulin resistance in muscle [41]. TINAGL1, decreased in CMT-*SORD* patient derived muscle, is an extracellular matrix component essential for normal muscle and capillary development [42]. THBS4, another extracellular matrix component, is increased in CMT-*SORD* patient muscle and known to be enriched at the neuromuscular junction [43] and to be increased upon denervation [44].

Several of the dysregulated proteins are localized to the endoplasmic/sarcoplasmic reticulum (ER/SR). Our electron microscopic analyses revealed mild dilatation of the SR, suggesting that the dysregulation of these proteins may impact on SR- morphology. However, it is important to emphasize that ER/SR alterations have also been previously associated with muscle denervation [45], primarily linked to disturbed calcium homeostasis, and therefore these changes should not be considered specific hallmarks of SORD deficiency. This is further supported by the fact that many of the ER/SR-resident proteins identified here are known to play roles in calcium regulation. Additionally, the proteomic signature showed dysregulation of numerous cytoskeletal proteins; data intersection analysis indicates that these changes are more likely consequences of denervation, a phenomenon well-documented in the literature [46]. Considering this metabolic rewiring in the broader context, one could speculate that these factors [47] may have contributed to the significant CK elevation to 13,000 U/L in patient 2 following physical exercise in a warm environment. This patient exhibited the highest levels of mutated *SORD* transcript according to our qPCR analyses, accompanied by consistently elevated expression of components of the complement cascade and immune response, as well as *NNMT*. Elevated NNMT levels in denervated muscle strongly suggest a catabolic, atrophy-associated state, potentially linked to impaired NAD^+^ metabolism. Impaired NAD^+^ metabolism is a recognized contributing factor in rhabdomyolysis because it compromises mitochondrial energy production and stress resistance, but it typically acts in combination with other stressors (such as heat) or underlying conditions, not as a sole trigger [48],[49]. These findings should be interpreted as supportive, but not definitive, evidence of disease-related muscle involvement. Further investigations are required to determine whether the observed degeneration is directly attributable to SORD deficiency. Importantly, a broader body of evidence, ideally encompassing multiple independent case reports, will be necessary to substantiate this hypothesis. Moreover, mild to moderately elevated CK levels have previously generally been reported in the context of muscle denervation [e.g.,[50], [51]].

## Conclusions

Our study demonstrates that SORD deficiency in skeletal muscle shares several hallmark features of denervation, including histological and ultra-structural changes as well as a broad pattern of protein dysregulations. However, distinct to SORD deficiency might be changes directly related to impaired sorbitol metabolism, such as metabolic rewiring involving altered glucose-to-fructose conversion, tetrapyrrole biosynthesis, and glycogen breakdown. While enhanced antioxidant responses and marked immune activation may result from this metabolic shift, they have also been previously associated with mitochondrial dysfunction in denervation. Together, our findings indicate that SORD deficiency combines classical denervation effects with unique metabolic alterations, offering starting points for further therapeutic avenues focused on restoring mitochondrial integrity and redox homeostasis. In this study, GDF-15 and FGF-21 have been excluded as informative minimal-invasive biomarker for CMT-*SORD*.

## Supplementary Material

Supplementary Table 2

Supplementary Materials

## Figures and Tables

**Figure:**
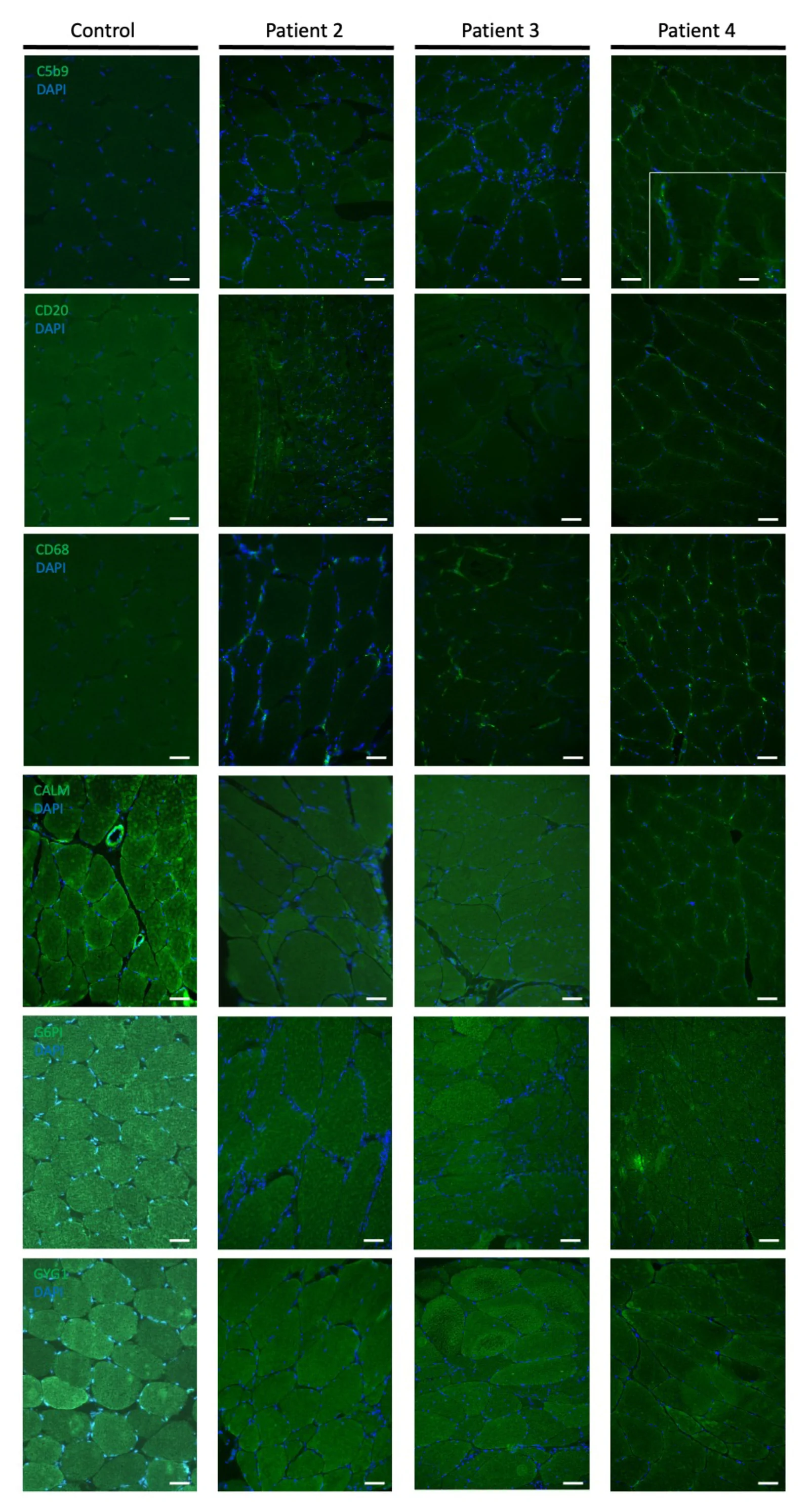


**Figure:**
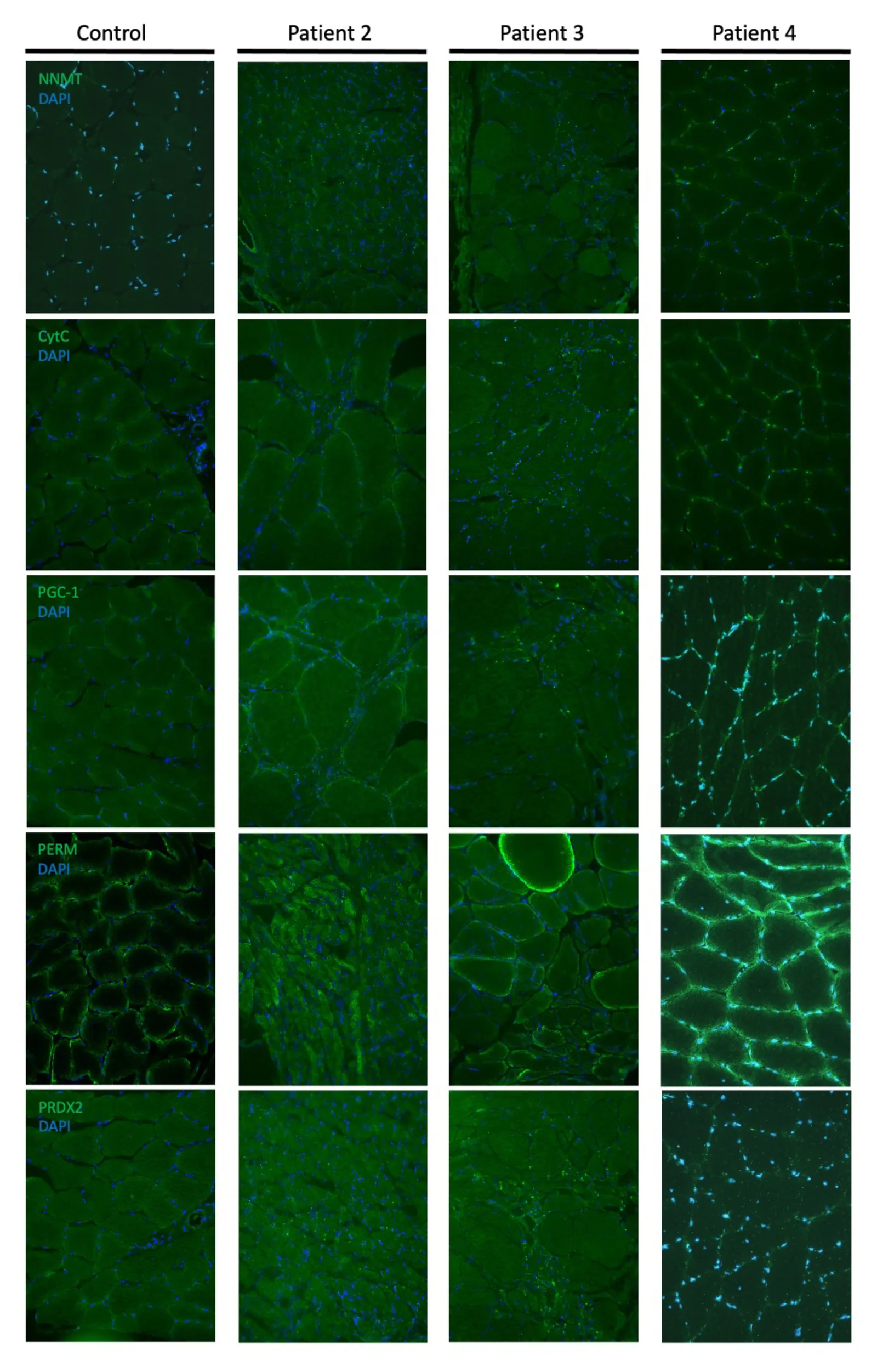


**Table 1:** Muscle biopsies and respective applied analytical applications in this study.

Gene	Patient	Muscle	Histology	Electron microscopy	Proteomics	Immunofluorescence
*SORD*	1		yes	no	yes	no
	2		yes	yes	yes	yes
	3		yes	yes	yes	yes
	4		yes	no	no	yes
*GAN*	5		yes	no	yes	yes
*GDAP1*	6		yes	no	yes	no

**Table 2:** Demographic, clinical, and genetic summary of four patients with SORD-associated CMT The table presents the demographic features, clinical presentation, neurophysiological findings, and genetic results of four affected individuals. All patients manifested distal-predominant motor neuropathy with variable sensory and biochemical abnormalities. Electrophysiological and histopathological studies confirmed axonal and neurogenic changes. Molecular testing identified the homozygous SORD c.757delG variant in two patients and pathogenic SORD variants in the remaining two.

FEATURE	PATIENT 1	PATIENT 2	PATIENT 3	PATIENT 4
**SEX**	Female	Male	Female	Female
**ETHNICITY**	Caucasian	Caucasian	Caucasian	Arabic
**FAMILY CONSANGUINITY**	No	No	No	Yes
**FAMILY HISTORY**	Unremarkable	Unremarkable	Unremarkable	Cardiomyopath y (father)
**AGE AT ONSET**	17 yrs	15 yrs	11 yrs	23 yrs (symptoms for 10 yrs before diagnosis at 33 yrs)
**INITIAL SYMPTOMS**	Foot weakness, burning paresthesia, itching after exercise	Lower limb weakness, foot drop, fatigue	Stumbling, gait unsteadiness, fatigue	Exercise-induced cramps, myalgia, fatigue
**PROGRESSION**	Gradual, involving proximal muscles; needs splints by 26 yrs	Gradual worsening to moderate distal flaccid paraparesis, foot dorsiflexion 3/5 (MRC) at age 27 years, pes cavus	Relapsing course; initial response to IVIG, steroids, plasmapheresis, lower-limb predominant moderate distal flaccid tetraparesis, foot dorsiflexion 3/5 at age 21 years	Mild progression, limited to distal lower limbs
**REFLEXES**	Brisk upper, normal knee, absent ankle	PTR reduced, ATR absent	ATR absent	Normal
**SENSORY** **FINDINGS**	Distal hypoesthesia (hands/feet)	None	Mildly reduced perception of light touch at lower legs	None
**CK LEVELS**	5023 U/L (high ↑)	Up to 800 U/L (mildly ↑), 1 exercise-induced rhabdomyolysis (CK 13.000 U/L)	Normal	~2× normal
**ELECTROPHYSIO** **LOGY (NCS/EMG)**	Distal axonal neuropathy → mild progression	Axonal, motor, leg predominant polyneuropathy (27 years)	Axonal, motor predominant polyneuropathy (21 years)	Normal NCS, mild neurogenic EMG
**MRI FINDINGS** **(AGE)**	Not specified	Fatty replacement + mild edema (calves)	Reticular + fatty calf changes	Asymmetric fatty degeneration (medial gastrocnemius)
**MUSCLE BIOPSY (AGE)**	Chronic partial denervation	Neurogenic pattern	Neurogenic pattern	Neurogenic pattern, atrophic fibers
**CSF**	Normal	Mildly ↑ protein	Normal	Normal
**GENETIC FINDINGS**	*SORD* c.757delG p.(Ala253GlnfsT er27) homozygous	*SORD* c.757delG p.(Ala253GlnfsT er27) homozygous	*SORD* c.757delG p.(Ala253GlnfsT er27) homozygous	*SORD* c.757delG p.(Ala253GlnfsT er27) homozygous
**COURSE SUMMARY**	Slowly progressive distal > proximal axonal neuropathy	Slowly progressive, axonal, motor polyneuropathy	Initially relapsing course, now slowly progressive motor predominant polyneuropathy	Mild distal neuropathy with preserved strength

**Table 3:** Comparative phenotypic severity across functional domains in patients with SORD-associated CMT The heat map summarizes the relative severity and clinical features of four patients carrying pathogenic *SORD* variants (patients 1-4). Each domain reflects a key aspect of disease manifestation, including motor weakness, sensory involvement, creatine kinase (CK) elevation, MRI abnormalities, neurogenic findings on muscle biopsy, and disease progression. Colour intensity indicates relative severity: ◯ = marked/severe, ◯ = moderate, ◯ = mild, ◯ = absent. All patients exhibited distal motor involvement and neurogenic biopsy changes, with variable sensory deficits, CK elevation, and MRI abnormalities. Patient 1 demonstrated the most severe and progressive course, whereas patient 4 presented with a milder phenotype and slow progression.

SYMPTOM	PATIENT 1	PATIENT 2	PATIENT 3	PATIENT 4
**MOTOR WEAKNESS**	◯	◯	◯	◯
**SENSORY LOSS**	◯	◯	◯	◯
**CK ELEVATION**	◯	◯	◯	◯
**MRI ABNORMALITY**	◯	◯	◯	◯
**BIOPSY NEUROGENIC PATTERN**	◯	◯	◯	◯
**PROGRESSION**	◯ Progressive	◯ Slowly progressive	◯ Relapsing at first, now slowly progressive	◯ Slowly progressive

Legend: ◯ = Marked, ◯ = Moderate, ◯ = Mild, ◯ = Absent, ◯ = Improvement

## Data Availability

The mass spectrometry proteomics data have been deposited to the ProteomeXchange Consortium via the PRIDE [52] partner repository with the dataset identifier PXD070751.
