# Supplementary material for: When Muscles Matter in SORD Neuropathy
Source: J Neuropathol Exp Neurol. Author manuscript; Available in PMC 2026 Nov 10. (PMC7619715; doi:10.1093/jnen/nlag057)
Supplement: Supplementary file 1 [file EMS222006-supplement-Supplementary_Materials.pdf]

Supplementary Figure 1 - Additional light microscopic characterization of CMT-*SORD* patient muscles: (**A & B**) Histology confirmed irregular mitochondrial distribution based on the NADH stain, but combined COX-SDH stain did not reveal COX-negative fibers. Immunohistochemical characterization of muscle biopsies revealed a mild increase in immunoreactivity for C5b-9, CD45, and CD68 in both a pediatric patient (patient 3; **A**) and an adult patient (patient 4; **B**). Additionally, a slight increase in CD3-positive cells was observed in the adult patient. (**C**) For comparison, staining for CD45, CD8, and CD68 in skeletal muscle biopsies from juvenile ASyS patients demonstrated consistent immunoreactivity.

Supplementary Figure 2 - Representative image of a semithin section of glutaraldehyde-fixed, resin-embedded muscle biopsy tissue, paraphenylenediamine stain: Note the groups of partially atrophic of atrophic, flattened or angular muscle fibers as well as hypertrophic fibers. Arrow: Necrotic muscle fiber invaded by macrophages, consistent with the ultrastructural findings shown in Fig. 3G-I. Scale bar = 20 µm.

Supplementary Table 1 - List of qPCR Taqman Assays used for quantitative PCR studies.

Supplementary Table 2 - List of significantly dysregulated proteins identified by mass spectrometry based proteomic profiling.

Supplementary Document 1

Patient 1: A female Caucasian patient from a non-consanguineous family with an unremarkable family history. The first symptoms appeared suddenly at the age of 17, characterized by weakness, burning paresthesia, and itching in the feet following exercise. Over the subsequent years, there was a gradual progression of weakness, including proximal muscles. By age 26, the patient required splints, and by age 30, she experienced further deterioration, including progressive weakness, muscle atrophy in the hands, and reduced physical endurance. Recurrent infections led to the removal of several toenails. Additionally, the patient developed muscle pain, which was exacerbated by physical activity, and pregabalin treatment provided no relief. At the age of 17 years, distal muscle weakness with bilateral foot drop (foot flexion/extension 3/5) was diagnosed. Hip flexion and knee flexion/extension were rated 4/5 on the MRC scale, as were wrist and finger extension/flexion. Distal hypoaesthesia was noted in the hands and feet. Reflexes were brisk in the upper limbs, the patellar tendon reflex was normal, but ankle reflexes were absent. She exhibited a slightly unsteady gait and was unable to walk on his heels or tiptoes. There were no signs of cerebellar, extrapyramidal, or cognitive dysfunction. Cerebrospinal fluid (CSF) analysis at the time of referral was normal, and creatine kinase (CK) levels were significantly elevated (5023 U/L), even after repeated measurements. Initial electrophysiological studies performed at age 24 years revealed distal axonal sensorimotor neuropathy. Over the following five years, her condition showed a slight progression, with worsening weakness and sensory symptoms in the feet and legs. Due to reduced exercise capacity and some impairment of neuromuscular transmission, the patient was started on pyridostigmine; however, no improvement was observed. Repeated nerve conduction studies (NCS) at age 33 years demonstrated a slight deterioration in motor and distal sensory conduction velocities, while needle electromyography (EMG) examination revealed chronic partial denervation in the vastus medialis and tibialis anterior muscles. Given the persistently elevated CK levels, a skeletal muscle biopsy was performed.

Whole genome sequencing performed in 2018 under the 100,000 Genomes Project, including analysis of the neuromuscular gene panel, did not identify a causative mutation. However, following the identification of *SORD* as a novel gene associated with axonal CMT disease, we requested a reanalysis of the patient's WGS data, which revealed that he carries the homozygous "common" *SORD* mutation, c.757del; p.(Ala253GlnfsTer27).

Patient 2: A male Caucasian patient from a non-consanguineous family with an unremarkable family history. At age 15 years, the patient experienced a sudden onset of progressive lower limb weakness, initially manifesting as difficulty climbing stairs—requiring the use of handrails—and foot dorsiflexion weakness. He also reported increased stumbling, decreased jumping power and sprinting speed during sports activities, along with an unsteady gait and pronounced fatigue, necessitating afternoon naps after school. Over the following six months, symptoms gradually worsened before reaching a stable phase. Prior to symptom onset, the patient had normal development and performed well academically. CSF analysis revealed mildly elevated total protein. MRI of the brain, spine, and both upper and lower limbs showed no abnormalities. Serum CK levels were repeatedly elevated, reaching up to 800 U/L. At the age of 28 years, the patient experienced one exercise- and heat-induced rhabdomyolysis with myalgia in thigh and lower back muscles and CK levels up to 13,000 U/l. NCS of the lower limbs demonstrated reduced amplitudes and prolonged latencies with preserved conduction velocities; F-waves were normal. Needle (EMG) of the tibialis anterior muscle indicated neurogenic remodelling. At age 16, a follow-

up MRI of the lower legs showed fatty replacement of muscle tissue alongside mild oedema. With myositis suspected, muscle and skin biopsies were performed from the left gastrocnemius muscle.

Whole exome sequencing revealed the homozygous c.757del; p.(Ala253GlnfsTer27) variant which causes a frameshift leading to a premature stop codon 27 residues downstream, in turn resulting in a truncated and likely nonfunctional protein.

Patient 3: A male Caucasian patient from a non-consanguineous family with an unremarkable family history. At age 12 years, following two episodes of influenza, the patient developed internal rotation of the right leg, increased stumbling, an unsteady gait, and marked fatigue requiring afternoon naps. Prior to this, her development was entirely unremarkable, with good academic performance. Initial NCS were within normal limits, though the F-wave response of the peroneal nerve was abnormal. EMG demonstrated a neurogenic pattern, while MRI of the spine and CSF analysis were unremarkable. Based on clinical suspicion of Guillain-Barré syndrome, intravenous immunoglobulin (IVIG) therapy was initiated, leading to gradual symptom improvement over six months. Following this period of improvement, the patient experienced a second deterioration, presenting with difficulty climbing stairs (requiring handrail support) and reduced jumping ability during sports. At this stage, repeat nerve conduction studies (including F-wave analyses of both upper and lower limbs) were unremarkable. Serum CK levels were within normal range. MRI of the lower limbs revealed pathological reticular and lipomatous changes in the bilateral calf muscles. Differential diagnoses at this point included metabolic or distal myopathy, with no electrophysiological evidence of chronic neuropathy. A muscle biopsy from the right gastrocnemius revealed a neurogenic pattern of muscle involvement. At age 16, a sural nerve biopsy was performed due to the limited and inconsistent response to immunotherapies, showing moderate chronic neuropathy of axonal type with a mild demyelinating component. NCS showed reduced amplitudes of motor nerves, predominantly affecting the lower limbs. Presence of axon reflexes was reminiscent of demyelinating neuropathies, however, we did not observe any conduction blocks. Nerve ultrasound showed very mild enlargements of nerve cross sectional areas (UPSS score 3/22 points) with normal diameters of the examined cervical nerve roots. After intervention with IVIG, further treatment approaches included corticosteroids, and most recently plasmapheresis, with the patient reporting subjective stabilization. The patient remains strongly motivated to pursue ongoing therapeutic intervention.

At the age of 19, re-evaluation of the trio-exome data with evidence of a mutation in the *SORD* gene. This re-evaluation revealed the homozygous c.757del; p.(Ala253GlnfsTer27) variant.

Patient 4: A female patient of Arabic origin, born to consanguineous parents, with a family history of hereditary cardiomyopathy in her father (autosomal dominant). The patient first presented at the age of 33 years with a 10-year history of exercise-induced myalgia, cramps, exercise intolerance, and fatigue. Past medical history was unremarkable apart from herpes virus–induced keratopathy requiring corneal transplantation at 20 years of age. On initial clinical examination, no muscle atrophy was evident. Sensation and tendon reflexes were preserved, and the remainder of the neurological examination was unremarkable. Nerve conduction studies, including F-wave analyses, were normal. Electromyography demonstrated a mild neurogenic pattern in the lower leg muscles. Serum creatine kinase was mildly elevated (approximately twofold). Cerebrospinal fluid and serological analyses were normal. Whole-body muscle MRI

revealed asymmetric fatty degeneration of the medial head of the gastrocnemius, while all other muscles appeared unremarkable. Muscle biopsy showed neurogenic alterations with large groups of atrophic fibers, nuclear bags, and fiber-type grouping.

Next-generation sequencing–based panel testing performed in 2019 did not identify any pathogenic or likely pathogenic variants. However, re-analysis in 2023 using whole-exome sequencing revealed the common pathogenic c.757del; p.(Ala253GlnfsTer27) SORD variant in homozygosity. At the most recent clinical evaluation, at 39 years of age, the patient exhibited mild atrophy of the intrinsic foot muscles, toe deformities, and weakness of toe flexion. There was no additional muscle weakness or atrophy. Tendon reflexes and sensory function remained preserved, and no additional neurological abnormalities were noted.

Supplementary Table 1

Antibody	Host	Dilution	Order ID	Firma
C5b9	Mouse/monoclonal	1:25	M0777	DAKO
CD20	Mouse/monoclonal	1:100	NCL-L-CD20-L26	Leica
PRDX2	Rabbit/monoclonal	1:20	MA5-32466	Invitrogen
CD68	Mouse/monoclonal	1:100	M0718	DAKO
G6PI	Mouse/monoclonal	1:100	Ab66340	Abcam
GYG1	Mouse/monoclonal	1:50	Sc-271109	SantaCruz
NNMT	Mouse/monoclonal	1:50	SC-376048	SantaCruz
CytC	Rabbit/monoclonal	1:100	Ab133504	Abcam
PGC-1	Rabbit/polyclonal	1:100	Ab54481	Abcam
PERM	Rabbit/polyclonal	1:50	HPA031712	SigmaAldrich/Merck
